# Media influence on humanitarian interventions: analysis of the Rohingya refugee crisis and international media coverage

**DOI:** 10.1186/s41018-021-00108-5

**Published:** 2021-10-16

**Authors:** Michelle J. Lee

**Affiliations:** Department of Population and Family Health, Columbia University, 722 W 168th St, New York, NY 10032 USA

**Keywords:** Rohingya, Refugees, News media, Humanitarian intervention, Foreign policy, Genocide

## Abstract

In 2017, the long-festering discriminatory treatment to the Rohingyas in Myanmar, both in law and practice, resulted in the largest cross-border humanitarian crisis in Asia. During the 2016‑2017 Rohingya refugee crisis, the aerial shots of burnt villages and images of people trudging toward the horizon in search of refuge in neighboring nations dominated the Western media. However, for humanitarians, the question of whether the media helps with humanitarian crises remains complicated and unclear. This study examines the effects of media coverage on the Rohingya refugee crisis based on articles from two liberal, elite newspaper sources, *The New York Times* and *The Guardian* between 2010 and 2020. The study reveals that the attempts of international pressure to stop the crisis have increased through media coverage and political pressures; however, the number of Rohingya refugees fleeing Myanmar intensified due to worsening violence and human rights violations committed by the Myanmar army. Findings are discussed using the lens of cultural and ideological context. The study suggests that in Myanmar, where authoritarian military culture is pervasive, there is a limited influence of the international press on the state-sponsored ethnic cleansing of the Rohingya population and questions whether consistent international pressure could have changed the outcome.

## Introduction

The Kutupalong camp in Bangladesh is the world’s largest refugee camp, with a population of almost one million Rohingya families (World Food Program 2020). The Rohingyas, a mostly-Muslim ethnic minority group in predominantly Buddhist Myanmar, are escaping historical prosecution and human rights abuses, both in law and practice (MSF 2017a, b). Despite travel restrictions on humanitarian agencies by the Myanmar government, the Rohingya refugee crisis gained significant global attention through graphic images, videos, and stories from the Rakhine State (Neuman 2018; Islam 2018). Furthermore, investigative reports published by reputable news outlets galvanized international attention to what the United Nations (U.N.) has called “a textbook example of ethnic cleansing” (OHCHR 2017; Mahmood et al. 2017; Vu and Lynn 2020). For humanitarians, however, the question of whether international media coverage helps with foreign policy and initiates humanitarian interventions to stop the refugee influx remains unclear and complicated.

Although there is no one standard or legal definition of humanitarian intervention, the Responsibility to Protect has been adopted since 2005 by the U.N., which states it would take timely and decisive action when national authorities of other countries manifestly fail to protect their populations from genocide, war crimes, ethnic cleansing, and crimes against humanity (United Nations 2005). There are four main different types of U.N. intervention action—assurance, diplomatic engagement, military involvement, and intimidation (Beardsley 2012), including humanitarian aid and international sanctions. Existing human rights literature shows that shaming tactics by human rights advocacy groups promote third-party actions against repressive regimes, such as reduced foreign direct investment (Barry et al. 2012), trade and economic sanctions (Peksen et al. 2014), and humanitarian armed interventions (Murdie and Peksen 2014).

The role of the foreign media and humanitarian interventions has been the subject of significant academic research, regarding the effects of news media on foreign governments’ political decisions to intervene, known as the ‘CNN effect’ (Robinson 2000; Balabanova 2010). The ‘CNN effect’ describes the policy-media interaction and predicts that media influence occurs when the policy is uncertain and media coverage is critically framed to empathize with suffering people, but when the policy is certain, media influence is unlikely to occur (Robinson 2000). Scholars often cite the 1992‑1995 Bosnian War (Robinson 2000) as an example of media influence on foreign policy; however, others remain skeptical, suggesting that the Bosnian example may be based on certain cultural and political assumptions, where “working with the same presumption outside of the West European context might not be very successful” (Balabanova 2010). Furthermore, recent literature on the “Amnesty international effect” has argued that human rights organizations’ activities appear to have a significant impact on the likelihood of military interventions led by third-party states (Murdie and Peksen 2014). Meanwhile, others have found that determinants of intervention decisions tend to focus on geopolitical and economic factors, often ignoring the potential non-state actors, such as media and humanitarian organizations, to influence foreign policy decisions on humanitarian intervention (Beardsley and Schmidt 2011; Choi 2013). In summary, scholarly and professional studies of the CNN effect that media has a direct effect in relieving humanitarian crises present mixed, contradictory, and confusing results (Gilboa 2005). Thus, for humanitarians, a question still exists on how the news media coverage trends affect the population group they report on and influence humanitarian intervention.

This study examines the effects of mass media coverage of the Rohingya refugee crisis that was driven by horrific images and narratives that served as a motivator for foreign policy action, including diplomatic humanitarian interventions and economic sanctions. In particular, this study complements the relevant literature on the possible link between global media coverage and humanitarian interventions by focusing on international diplomatic changes on the causes of the crisis to stop it. By examining news media coverage of the Rohingya crisis in two elite newspaper sources, including *The New York Times* and *The Guardian*, this study aims to compare the media coverage trends to the displacement rates of Rohingya refugees between January 1, 2010, and January 1, 2021. Establishing this timeline allows us to compare variation in the frequency of news reports to trends of forced displacement from Myanmar reported to the United Nations High Commissioner for Refugees. The questions guiding this study were (1) How did media coverage of the two newspapers on the Rohingya refugee crisis change between 2010 and 2020? (2) How do media trends correspond to the number of Rohingya refugees? (3) How do media trends correspond to key policy changes and humanitarian interventions by the United Nations and other humanitarian agencies to stop the causes of refugee influx?

International media attention is an important tool to start the process of mobilization and opinion-shaping of the international community by shining light on egregious human rights violations in remote parts of the world. This study argues that increased media attention to the Rohingya refugee crisis increases the likelihood of humanitarian intervention to reduce the number of Rohingya refugees fleeing from violence in Myanmar. As the number of media coverage increases, this would mobilize the international pressures to stop the causes of the refugee influx and potentially initiate various humanitarian interventions that aim to protect the human rights of Rohingyas in Myanmar.

To better contextualize and provide a holistic understanding of this study, the paper will first examine the historical background of the Rohingya refugee crisis. Thereafter, the paper will describe the methodological framework employed in this study to analyze the available data from the United Nations High Commissioner on Refugees (UNHCR) and application programming interface (API) from *The Guardian* and *The New York Times.* This will be followed by a discussion of the findings of the study. The paper now turns to examine the historical context of the Burmese-Rohingya conflict.

### The Rohingya crisis

Living in a country where almost 90% of the citizens are Buddhist, Rohingyas have been dehumanized by socio-historically constructed and imposed identity throughout the colonial and post-colonial years by the majority Buddhists (Siddiquee 2019). They are perceived as illegal and unwelcome foreigners, as people call them “Bengalis” and reject using the term “Rohingya” to deny them of their ethnic heritage (Rosenthal 2019). One of the major systematic violations against the Rohingyas is the discriminatory 1982 citizenship law, which disenfranchised the Rohingya people from equal access to full citizenship in Myanmar, leaving one in seven stateless persons in the world as Rohingya ever since (Tran 2015; Mahmood et al. 2017; MacLean 2019). Another notable example of systematic erasure is the U.N.-backed 2014 census, in which the government banned the term “Rohingya” and replaced it with “people who believe in Islam in Rakhine State,” effectively uncounting more than one million people from the national census (Ferguson 2015; Southwick 2018; MacLean 2019).

Furthermore, the Rohingyas have been systematically denied access to fundamental social services such as education, health care, reproductive health, and the internet, effectively limiting them from access to critical information and aid from international humanitarian organizations (Khin 2017; Rosenthal 2019; Bakali and Wasty 2020). Before 2017, more than 70% of the Rohingya lacked access to safe water and sanitation services, and only 2% of Rohingya women gave birth in hospitals (Khin 2017).

In addition to decades of systematic discrimination, persecution, and statelessness, the Rohingyas have faced waves of violence between 1978 and 2017 that have forcibly displaced them into Bangladesh (Baird 2020). The 2017 Rohingya refugee crisis is not an isolated event; rather, it is part of a series of major military operations targeting the Rohingyas by the state’s armed forces, the *Tatmadaw* (MacLean 2019). In 1978, Operation Dragon King (*Nagamin*) sought to take action against ‘illegal immigrants,’ forcing more than 200,000 Rohingya refugees to Bangladesh as a consequence of the violence and human rights abuses during its operations by the *Tatmadaw* (Khan and Munshi 1983; MacLean 2019). In 1992, another round of operations forced more than 300,000 Rohingya refugees into Bangladesh, with horrifying accounts of rape, forced labor, and religious persecution (Human Rights Watch 1993; Toole and Waldman 1997).

In October 2016, the *Tatmadaw* launched a major crackdown on the Rohingya, including men and boys being taken for forced labor, girls and young women raped and sexually exploited, and children disappearing (United States Department of State 2016). Notably, the *Tatmadaw* adopted Facebook to promote anti-Rohingya propaganda online, in which hundreds of military officials created fake names, news, and entertainment pages and then posted inflammatory posts portraying Rohingya as terrorists (Mozur 2018). Without much accountability, Facebook continues to be the platform where *Tatmadow* officials spread hate speech and disinformation against the Rohingya people, causing “a negative impact on freedom of expression, assembly and association for Myanmar’s most vulnerable users” (Stevenson 2018).

Triggered by a combination of intensifying violence, human rights violations, and systematic marginalization, the major exodus of Rohingya refugees began in October 2017 (Sida 2018), as more than 647,000 Rohingya fled to Bangladesh while 6700 were killed during the violent attacks (MSF 2017a, b). International organizations documented large-scale atrocities against civilians by the *Tatmadaw* including murder, torture, rape, and wanton destruction of property (Akhavan 2019), which the U.N. (OHCHR 2017) and Amnesty International (2018) described as “ethnic cleansing” and “genocide.” In January 2020, the International Court of Justice in The Hague unanimously ordered Myanmar to take all necessary measures to prevent the genocide of the Rohingyas in Myanmar (ICJ 2020), but little is known about the effects of the order on improving the living conditions of the Rohingyas in Myanmar.

Despite strong advocacy by the U.N. and international humanitarian agencies for unhindered humanitarian access, the Myanmar government has banned humanitarian organizations and their staff to visit the Rakhine State, restricting the U.N. from verifying reports of ongoing human rights violations and the number of people still internally displaced or fleeing (OCHA 2017). This “information blackout” raises urgent humanitarian needs of Rohingya still in Myanmar and 128,000 people who are still confined in camps for internally displaced people (OCHA 2017). About 78% of them are women and children, who live in “overcrowded shelters and inadequate access to services and living opportunities” and their well-being continues to remain unknown and invisible to international observers (OCHA 2017; MacLean 2019).

The scale and speed of displacement were unprecedented in both Bangladesh and the wider region, creating significant humanitarian needs and impacting host communities (UNDP 2018). Although surrounding countries, such as Bangladesh, and international organizations have been compassionately aiding to alleviate the consequences of the Rohingya refugee crisis, humanitarian intervention policies should aim to act on the causes of the crisis to stop it, as it is not sustainable for other countries to continue hosting fleeing Rohingyas. Throughout the refugee camps in Bangladesh, the COVID-19 pandemic has worsened the living conditions, made access to services even more challenging, increased the risk of sexual and gender-based violence, and exacerbated the impacts of infectious diseases in crowded camps (UNHCR 2020). Lack of access to critical and life-saving services, such as food, drinkable water, latrines, and limited access to health services are turning an already serious crisis into a major human disaster (Banik and Rahman 2020). In summary, forced displacement, segregation, and severe restrictions on freedom of movement and press all contribute to “creeping apartheid,” a form of ethnic cleansing in slow motion, that makes it “easier to carry out large sale clearance operations” (MacLean 2019).

Having discussed the conditions that created the Rohingya refugee crisis, the paper now turns to describe the methodological frameworks and processes employed in this study to examine media influence on international humanitarian aid to the Rohingya refugee crisis.

## Materials and methods

The purpose of this article is to capture media coverage of the Rohingya refugee crisis in the USA and the UK, to explore the coverage trends and their influence on humanitarian intervention. We selected newspapers that combined both high readership and quality. Two news sources, *The New York Times* and *The Guardian*, are on the list of newspapers in the USA and the UK as top newspapers by circulation. *The New York Times* was selected because it ranks third in the USA with the highest circulation (Agility 2021), covers international issues extensively (Vu and Lynn 2020), and is renowned for its reputable investigative journalism. We did not select the newspaper with the first- (*New York Post*) and second-highest circulation (*The Wall Street Journal*), because the *New York Post* is a classic tabloid format with a focus on entertainment, and *The Wall Street Journal* is primarily toward business news rather than general news (Kupchik and Bracy 2008). It is noteworthy that *The New York Times* is a liberal media outlet (Vu and Lynn 2020) and does bias our sample toward reports geared to readers in the Northeastern United States (Kupchik and Bracy 2008). *The Guardian* is Britain’s leading center-left quality newspaper and one of the country’s most popular news websites, with a total daily reach of more than 4.1 million people (Guardian 2018; UNHCR 2015). It attracts an elite audience and also has a reputation for the quality of its investigative journalism.

The timeframe is between January 1, 2010, and January 1, 2021 (11 years). This timeframe was chosen to observe the years leading up to and after the most recent crisis that triggered a mass exodus of Rohingyas in August 2017. Establishing this timeline allows us to compare variation in the frequency of news reports to trends in reported migration, which we obtained from the United Nations High Commissioner for Refugees. The figures that we cite come from the Refugee Data Finder from UNHCR, and using key terms population figures (dataset), demographics (display type), all population types (forcibly displaced population, stateless persons, others of concern to UNHCR), Myanmar (country of origin), and Bangladesh (country of asylum) between 2010 and 2021.

Data from *The New York Times* and *The Guardian* were retrieved via each newspaper’s open data Application Programming Interface (API) service. We created a code using the R programming language to search and retrieve article information (published date, title, URL, and type), using “Rohingya” as the search term. We included news materials that were fact based, such as the world, New York, Business Day, multimedia, photos, U.S. technology, podcasts, but eliminated opinions, daily briefings, and culture (book, movie, arts, style, sports) reviews. This screening process limited our sample to articles written by professional journalists covering the Rohingya refugee crisis. After eliminating duplicates, the final samples included 488 articles from *The New York Times* and 814 articles from *The Guardian.* Processing was conducted to delete all the irrelevant categories (status, copyright, lead paragraph, print section, print page, document type, news desk, subsection name, id, word count), and only headlines, snippet, URL, section name, and date were retained.

## Results

Since only yearly records of the Rohingya refugees were available, the number of online articles covering the Rohingya refugee crisis by *The New York Times* and *The Guardian* were also consolidated by year to match the UNHCR dataset. Table 1 shows that between 2010 and 2015, the number of Rohingya refugees fleeing Myanmar was consistent at around 230,000 people, which was in response to escalating military-driven campaigns that scrutinized their citizen status and violent efforts to expel illegal foreigners (Rahman 2015). In 2015, the images of Rohingyas fleeing Myanmar on dangerous boat voyages that drowned at least hundreds of people captured international attention (Southwick 2018; Rosenthal 2019), as media coverage of the Rohingya refugee crisis increased by 240% by *the Guardian* and 116% by *the New York Times* from the previous year. However, in 2016, there was a sharp decrease in media coverage as articles related to the crisis decreased by 62% by *the New York Times* and 48% by *the Guardian*.

**Table 1:** # of article frequency and Rohingya refugee by year

*Year*	*Refugees*	*Refugees change (%)*	*New York Times*	*NYT change (%)*	*The Guardian*	*The Guardian change (%)*
2010	229226	NA	4	NA	1	NA
2011	229644	0.18	0	−100.00	0	−100.00
2012	230674	0.45	18	Inf	30	Inf
2013	231125	0.20	19	5.56	27	−10.00
2014	232462	0.58	30	57.89	25	−7.41
2015	231948	−0.22	65	116.67	85	240.00
2016	276198	19.08	25	−61.54	44	−48.24
2017	932204	237.51	127	408.00	174	295.45
2018	906635	−2.74	88	−30.71	229	31.61
2019	854764	−5.72	60	−31.82	93	−59.39
2020	860416	0.66	51	−15.00	106	13.98
Total	5215296	NA	487	NA	814	NA

In 2017, there was almost a threefold increase of Rohingya refugees to 932,204, in which *The New York Times* published its highest number of articles (127), while *The Guardian* did so in 2018 with 229 articles. This is best explained by the latest crisis that began on August 25, 2017, when allegedly an armed Rohingya organization (the Arakan Rohingya Salvation Army) attacked police stations and government officials (Human Rights Watch 2017; Khin 2017; Southwick 2018). In a series of events that the U.N. described as a “textbook example of ethnic cleansing” (OHCHR 2017), the military retaliated by indiscriminately targeting civilians as they burned villages, shot children and women, gang-raped women, looted, and blocked humanitarian aid (Khin 2017; Revesz 2017; Gettleman 2017). Evidence suggests that there had been troop build-ups and heightened military-sponsored Islamophobia for weeks prior, suggesting that the military operations were pre-planned to drive most of the Rohingya out of the country (Khin 2017; Fortify Rights 2017; Southwick 2018).

**Fig. 1:**
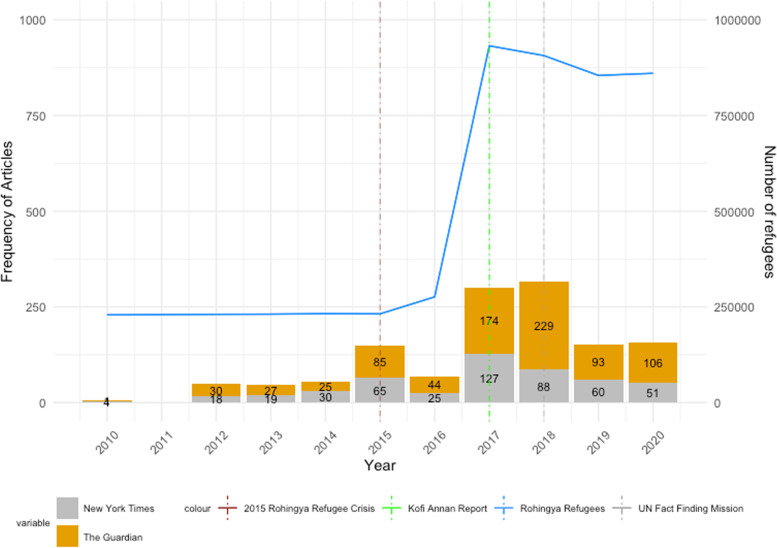
Frequency of news articles, number of Rohingya refugees

In Fig. 1, we present the yearly trends of the Rohingya refugees fleeing Myanmar and the media coverage of the Rohingya refugee crisis between 2010 and 2020. Three vertical lines represent significant international events related to the Crisis. The ‘2015 Rohingya Refugee Crisis’ indicates the well-publicized plight of the “boat people,” as a result of irregular migration flows of Rohingya from Rakhine State to neighboring countries in barely seaworthy boats (Rosenthal 2019), but the media coverage declined sharply in 2016. On August 24, 2017, the Advisory Commission on Rakhine State, which was led by the former U.N. Secretary-General Kofi Annan and institutionalized by Myanmar, published its final report on humanitarian issues related to the Rakhine State (Vu and Lynn 2020). Lastly, on September 12, 2018, the United Nations Fact-Finding Mission on Myanmar published its final report on the human rights concerns of the Rohingya population (OHCHR 2018a, b).

In Fig. 1, we see that the first notable trend was between 2014 and 2016 when the Crisis started to attract significant international media coverage in 2015. Between 2015 and 2016, despite a slight increase in the number of Rohingya refugees fleeing Myanmar, the media coverage significantly decreased, showing inverse trends. The sharp increase in media coverage of the Rohingya refugee crisis in 2015 indicates an ominous prelude to what was to come in August of 2017, as violence and discrimination intensified in Myanmar. Then, the number of Rohingya refugees spiked in 2017, along with *The New York Times* and *The Guardian.* Since 2018, the number of Rohingya refugees has remained steady at around 850,000, while the media coverage trends have decreased significantly. In conclusion, as Fig. 1 illustrates, the study found that the western media coverage did not have a substantial effect to reduce the number of Rohingyas fleeing violence in the Rakhine State.

**Fig. 2:**
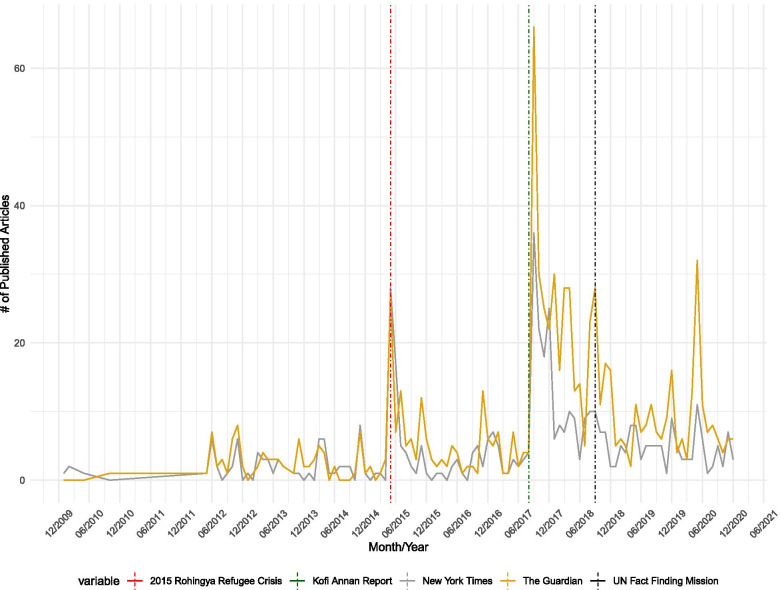
Media coverage trends by NYT vs. The Guardian between 2010‑2021 by month

In Fig. 2, we present the number of news articles published online each month in *The New York Times* and *The Guardian* that contained the term “Rohingya.” The media coverage trends by the two news outlets are comparable, as the number of publications per month increased and decreased during the same time throughout 2010 and 2020. Between January 2010 and April 2015, there was minimal international media coverage, as both news outlets published fewer than ten articles per month. In May 2015, *The New York Times* published 27 articles while *The Guardian* published 28 articles. This marks the first uptick in media coverage trends, considering that *The New York Times* published zero and *The Guardian* published two articles just a month before. Then, the media coverage trends decreased to around ten articles per month between August 2015 and August 2017. It then spiked again in September 2017, in which *The Guardian* published 66 articles while *The New York Times* published 36 articles, indicating the highest peak in Fig. 2.

In summary, our exploration in Figs. 1 and 2 indicates no evidence that during the study period the number of Rohingya refugees fleeing from violence in Myanmar decreased, as the number of global media attention increased and investigative reports by the United Nations were published. While there is a slight decrease in the number of refugees leaving since the publication of the reports in 2017 and 2018, the number remains still too high to conclude that there was some media coverage influence on the Rohingya refugee crisis to deter ongoing human rights violations and violence in Myanmar. Furthermore, the media’s reporting has substantially waned since 2018, whereas the number of Rohingya refugees fleeing remains fairly high.

## Discussion

The Rohingya crisis serves as an interesting case to compare the media coverage trends and how they correspond to the number of Rohingyas fleeing Myanmar, in the context of a pervasive authoritarian culture that defines its army (Rosenthal 2019). Our analysis unveils that the effect of global media on humanitarian aid might not be as effective as humanitarians would have hoped to deter military-driven genocidal campaigns against the Rohingyas and the empirical evidence suggests that there was no direct and long-term effect of decreasing the number of Rohingya refugees despite increased international media reports and diplomatic pressure.

Since the events in August 2017 that the U.N has cited as “bear(ing) the hallmarks of genocide” (OHCHR 2018a, b), horrifying personal accounts and the sheer scale of brutal human rights violations immediately mobilized international media attention (Rosenthal 2019; MacLean 2019). The aerial shots of burnt villages and images of people trudging toward the horizon in search of refuge in neighboring nations dominated the media, as the number of online publications increased by 408% for *the New York Times* and 295% for *The Guardian* (Table 1). However, the number of Rohingya refugees fleeing Myanmar continues to stay high around 850,000 per year (Table 1), despite official investigative reports by the Kofi Annan Foundation (Kofi Annan Foundation 2017) and the U.N. Fact-Finding Mission (OHCHR 2018a, b). The findings do not support the study hypothesis, and the study concludes that little evidence exists to support the claim that global media coverage influences humanitarian intervention actions or any changes in the Myanmar government that would stop the causes of the crisis and reduce the number of Rohingya refugees fleeing from the violence inflicted by the *Tatmadaw*.

The U.N. Special Rapporteur on the situation of human rights in Myanmar, Yanghee Lee, has questioned “whether the UN and international community could have prevented or managed the situation differently that occurred regarding the Rohingya and in Rakhine State” (OHCHR 2018a, b). Scholars have criticized the lack of significant consistent high-level pressure put on Myanmar to end egregious human rights violations and highlighted muted responses from global leaders (Khin 2017). On October 7, 2016, during the height of the *Tatmadaw*’s clearing operations in the Rakhine State, the Obama administration lifted sanctions against Myanmar “to support efforts by the civilian government and the people of Burma to continue their process of political reform and broad-based economic growth and prosperity” (U.S. Department of the Treasury 2016; Khin 2017; Southwick 2018). Furthermore, foreign aid and investment in Myanmar dramatically increased to USD 6.6 billion between 2016 and 2017, in which the USA and Britain were among the top investors (Zaw 2018). While recent studies have found that exposure of human rights abuses through news media increases the likelihood of economic sanctions imposed by the USA, research on the effects of economic sanctions remains unclear (Peksen et al. 2014).

Since October 2017, the USA, the UK, and the E.U. have suspended or reduced military assistance to Myanmar units linked to violence against the Rohingya and sanctioned military officials (The Guardian 2017; Peel 2017). Nonetheless, these sanctions do little to “address the underlying causes of the conflict in Northern Rakhine State, which range from chronic underdevelopment and growing competition over the scarce resource to institutionalized discrimination and Islamophobia” (MacLean 2019; Appadurai 2018). Scholars have strongly urged for the U.N. mandated global arms embargo to ban investment with military-owned companies in Myanmar, visa-bans, and support a referral of the situation to the International Criminal Court (Khin 2017).

One reason that the number of refugees did not diminish, despite increased international attempts to exert pressure for the end of the crisis, could be the lack of openness of the Myanmar regime to pressures. In the case of the Rohingya refugee crisis, despite international attempts to end the crisis by media outlets and international aid organizations, the results to reduce the number of refugees fleeing Myanmar remained poor. Past studies have found that when state-sponsored genocides have already begun, the perpetrators likely have already evaluated the international context and decided that there is sufficient permissiveness to commit genocide without consequence, in which merely signaling that the world is watching is likely to do little to stop genocides (Krain 2005). For example, the Myanmar army might have perceived the sharp decline of international media coverage of the crisis in 2016 as a loss of international interest and awareness of the atrocities in Myanmar. This observation raises the question of whether consistent escalation of international media coverage and pressure starting from 2015 could have altered the outcome in 2017 and affects the decision to intervene.

While current literature remains limited on motivations and effectiveness of humanitarian interventions in stopping genocides, a recent study suggests that interventions that directly challenge the regime are the only effective type of military response to slow or stop state-sponsored genocides, rather than ineffective impartial interventions (Krain 2005). Similarly, non-military responses, such as media coverage and campaigns by aid organizations, had little impact on the genocidal mission of Myanmar’s military rulers. In fact, scholars have found that interventions can shorten a conflict, but might hasten perpetrators to ramp up their genocidal policy within that period of time. This pattern is found in Myanmar, where the overt military mission of ethnic cleansing started in 2015 and its severity increased sharply between 2017 and 2018, after the rise of international pressure and interest. Future policies should focus on directly restraining or disarming the military and removing them from power to alleviate atrocities against Rohingyas in Myanmar (Krain 2005).

Another reason for the lack of direct military intervention to reduce the number of Rohingya refugee influx lies in the motivations to intervene and competing for geopolitical interests. As recent history shows, less than humanitarian motivations to intervene have successfully slowed or stopped state-sponsored mass killings (Power 2002; Krain 2005). For example, humanitarian intervention by India in East Pakistan, in what is now Bangladesh, was primarily an effort to counter Pakistani power and to stem the tide of refugees, and only secondarily a humanitarian mission to protect the ethnic Bengalis during the 1971 civil war in Pakistan (Charney 1999). Similarly, noble and humanitarian aims by international media and aid organizations to intervene in Myanmar did not translate into effectiveness, and unfortunately, there is a little political will to spur intervention and multilateral support as China and Russia hold permanent membership on the United Nations Security Council (Kingston 2015; Kichmann 2020). Furthermore, there are other pressing human rights crises attracting international attention in Asia, including the state-sponsored persecution of Uighurs in China. Both Uighurs and Rohingyas are Muslim ethnic minorities who are facing abuses at the hands of China and Myanmar. Recent studies have found that China’s position as a hegemony makes the cost of interventions outweigh any potential material or political benefit for the intervenors (Kichmann 2020). Intervention and assistance from Muslim organizations and Muslim-majority nations are encouraged by the current literature; however, they have largely remained relatively silent, prioritizing economic and strategic relationships with China (Maizland 2019). While studies often rely on non-violent international pressure to cease ethnic cleansing, such as campaigns by the Human Rights Watch and judgments from the International Court of Justice (Kichmann 2020), the findings from this study indicate that international media does little to increase international pressure to influence foreign policies aiming to reduce the number of Rohingya refugees or to stop the causes of the refugee crisis.

The lack of access to Rakhine State for humanitarian agencies due to the travel ban limits the scope of this study, as precise and detailed information of the ongoing human rights violations are unavailable online. The UNHCR website only offers yearly statistics of those who are registered as refugees from Myanmar and settled in Bangladesh refugee camps, excluding a number of those who have settled in other countries, those living in host communities, or certain locations beyond the camp boundaries, died en route to Bangladesh, and those who were killed in Rakhine State (UNHCR 2021). The precise number of those who were affected by escalating violence in the Rakhine State remains unknown.

This study could be strengthened by examining the content of published articles to understand how the Rohingya crisis is described and framed to influence the public and shape a country’s foreign policies toward humanitarian interventions (Vu and Lynn 2020). Future studies can assess textual elements to classify the content of the articles, such as narration of the crisis or narration of the humanitarian responses to the crisis, to identify precise media coverage trends related to the articles on the causes of the crisis and the ones acting on the consequences of the crisis.

Also, future studies can collect geospatial level and monthly-level data of refugee flow and analyze time-series panel regression models to identify the factors most closely associated with refugee flow and media reports. This would be possible when more detailed investigations are performed in Rakhine State, and statistics of Rohingya refugees become available after the ban of humanitarian agencies is lifted. Furthermore, we studied only two newspapers, which we chose based on their API availability and their readership. Future studies can include more newspapers located in other parts of the USA or the UK, or the world. It would be also interesting to examine how other media sources, such as television news and radio, may produce different results.

## Conclusion

This study contributes to a growing literature on media-foreign policy interaction by comparing the number of Rohingya refugees fleeing Myanmar to the frequency of articles published by two of the most widely circulated newspapers in the USA (*The New York Times*) and the UK (*The Guardian*) between 2010 and 2021. Despite substantial growth in media coverage and U.N.-backed investigations in Myanmar, little evidence exists to support the claim that exposure of human rights abuses through the news media increases the likelihood of humanitarian intervention against Myanmar to reduce the number of Rohingya refugees. The 2016‑2017 Rohingya refugee crisis is not an isolated event, rather, it is the climax of festering military operations that targeted Rohingya throughout the past half-century (Amnesty International 2004; MacLean 2019). The excruciating question of how to best serve and protect so many refugees concentrated in overcrowded camps is not just for Bangladesh or Myanmar, it is a question faced by the international community in general (Rosenthal 2019). Our study demonstrates that the exposure of human rights abuses against the Rohingya crisis through news media is essential to start mobilization of humanitarian agencies, but not sufficient to initiate humanitarian interventions to significantly reduce the number of refugees fleeing violence from Myanmar. Other consistent and high-pressure diplomatic interventions appear to be necessary to initiate changes that will protect Rohingyas’ ethnic heritage, collective identity, and the physical, and mental well-being of this vulnerable population.

## Data Availability

The datasets generated and analyzed during the current study are available in the “nyt_guardian_rohingya_dataset” repository, [https://github.com/mjl2241/nyt_guardian_rohingya_dataset].
